# Identification of Key Genes Associated with 1,2,6-Tri-O-galloyl-β-D-glucopyranose Accumulation in *Camellia sinensis* Based on Transcriptome Sequencing

**DOI:** 10.3390/foods13030495

**Published:** 2024-02-04

**Authors:** Yueqi Wang, Hanshuo Xun, Liubin Wang, Shirin Aktar, Yuping Lei, Rui Zhang, Liyuan Wang, Kang Wei

**Affiliations:** Key Laboratory of Biology, Genetics and Breeding of Special Economic Animals and Plants, Ministry of Agriculture and Rural Affairs, Tea Research Institute Chinese Academy of Agricultural Sciences, Hangzhou 310008, China; wangyueqi@tricaas.com (Y.W.); xunhanshuo77@126.com (H.X.); wangliubin@tricaas.com (L.W.); aktershirin1992@gmail.com (S.A.); leiyuping@tricaas.com (Y.L.); 82101215162@caas.cn (R.Z.); wangly@tricaas.com (L.W.)

**Keywords:** *Camellia sinensis*, transcriptome sequencing, UDP glycosyltransferase, serine carboxypeptidases, 1,2,6-TGGP

## Abstract

Hydrolyzed tannin 1,2,6-tri-O-galloyl-β-D-glucopyranose (1,2,6-TGGP) possesses significant medicinal properties. However, little is known about its underlying molecular mechanisms. In this study, the levels of 1,2,6-TGGP in tea materials from different cultivars and leaf positions were compared. Additionally, one leaf and one bud sample from six tea cultivars with significant variations in 1,2,6-TGGP levels were analyzed using transcriptome high-throughput sequencing to identify the genes that are responsible for 1,2,6-TGGP accumulation. The sequencing results were mapped to the reference tea genome, revealing a total of 2735 differentially expressed genes (DEGs). This set included four UDP glycosyltransferase (UGTs) and six serine carboxypeptidases-like (SCPLs) genes. Among them, the upregulated SCPLs (*CSS0032817*) may directly participate in the acylation reaction of 1,2,6-TGGP. In addition, several classes of DEGs, including cytochrome P450, were significantly associated with the 1,2,6-TGGP content, which is potentially involved in their regulation. Overall, these results provide new insights into the molecular mechanism of 1,2,6-TGGP accumulation.

## 1. Introduction

Hydrolyzed tannin 1,2,6-tri-O-galloyl-β-D-glucopyranose (1,2,6-TGGP) is a naturally occurring hydrolyzable tannin that was first identified in *Cornus officinalis* via cell culture in 1989 [1]. It has since been widely recognized for its anti-inflammatory and bacteriostatic properties. Duan et al. (2004) isolated 1,2,6-TGGP from extracts of traditional medicinal herbs and discovered its significant inhibitory effect on the hepatitis C virus (HCV) NS3 protease [2]. Kim et al. (2009) investigated the anti-inflammatory properties of seven natural gallotannins and determined that 1,2,6-TGGP exhibited the most effective inhibition of the lipopolysaccharide-induced inflammatory reaction [3]. Bag et al. (2013) isolated 1,2,6-TGGP from *Terminalia chebula* fruit [4]. Further studies have found that 1,2,6-TGGP exhibits inhibitory activity against multidrug-resistant (MDR) uropathogenic bacteria in the urinary system, particularly after the use of antibiotics, and its antibacterial effectiveness increased from 21.87% to 93.40% [5,6]. According to these reports, 1,2,6-TGGP could be used as an active compound in the healthcare and pharmaceutical industries. Therefore, 1,2,6-TGGP has been attracting increasing attention.

Tea is one of the most widely consumed non-alcoholic beverages in the world. It is rich in various functional secondary metabolites, such as tea polyphenols, which are beneficial to health [7]. Hydrolysable tannin 1,2,6-TGGP, a polyphenolic compound, was initially identified in green and fermented teas in 2008 [8,9]. Afterward, several scientists have also discovered 1,2,6-TGGP while studying polyphenols in tea plants [10,11,12]. We previously identified and verified 1,2,6-TGGP in tea plants using ultra-performance liquid chromatography–quadrupole-time-of-flight mass spectrometry, electrospray ionization mass spectrometry, and nuclear magnetic resonance. We found that the contents of 1,2,6-TGGP in different cultivars varied significantly [13]. This raised our curiosity about the potential mechanisms that affect the accumulation of 1,2,6-TGGP in tea plants.

The basic biosynthesis of 1,2,6-TGGP has been extensively reported. It proceeds through a series of strictly position-specific galloylation steps, after which the gallic acid (GA) is transformed into 1-O-galloyl-β-D-glucose (βG), 1,6-digalloylglucose, and finally, 1,2,6-TGGP [8,13]. The enzymes that catalyze the formation of βG from GA and UDP glucose belong to the UGT84A family and have been identified in various plants, including *Juglans regia* [14], *Quercus robur* [15], *Punica granatum* [16], and *Canarium album* [17]. Cui et al. (2016) also identified the key enzyme CsUGT84A22 as being involved in the biosynthesis of βG in tea plants, which facilitates the synthesis of ester catechins [18]. This suggests that βG is a common substrate for the biosynthesis of hydrolyzed tannins and ester catechins. However, it is unknown whether this enzyme is the key to the accumulation of 1,2,6-TGGP. Furthermore, specific genes that are involved in the 1,2,6-TGGP synthetic pathway have not been reported.

Transcriptome sequencing offers comprehensive and rapid access to nearly all transcripts of a species in a specific state through high-throughput sequencing of tissues in various physiological states and under different environmental conditions. This method reflects the expression of genes in cells and their overall regulation. In recent years, RNA-Seq technology has been widely utilized to identify candidate genes that are linked to the biosynthesis of secondary metabolites in tea plants [19,20,21]. To investigate the key genes that influence the synthesis of 1,2,6-TGGP, this experiment determined the 1,2,6-TGGP content and performed transcriptome sequencing analysis on different resources. It aims to screen and identify key candidate genes that may be involved in 1,2,6-TGGP accumulation and provides a theoretical basis for future research on the mechanisms of 1,2,6-TGGP accumulation and the breeding of high 1,2,6-TGGP tea cultivars.

## 2. Materials and Methods

### 2.1. Plant Materials

The tea cultivars ‘Zhongming 6’ (ZM6), ‘Zhongming 66’ (ZM66), ‘Zhongcha 108’ (ZC108), ‘Zhongcha 102’ (ZC102), ‘Longqu 1’ (LQ1), and ‘Wuniuzao’ (WNZ) were cultivated in the experimental tea garden of the Tea Research Institute, Chinese Academy of Agricultural Sciences, Hangzhou, China (120°12′ E, 30°16′ N). Fresh plant materials (one bud and one leaf) were collected and stored at –80 °C from 15 March to 27 March 2022. The tea cultivar ‘WNZ’ is the earliest to sprout, around 15 March, followed by ‘ZC102’, ‘ZC108’, ‘ZM6’, and ‘ZM66’, which sprout around 22 March. ‘LQ1’ is the latest to sprout, around 27 March. In April, one bud and one leaf, the third leaf, the fifth leaf, and mature leaves from the one bud and five leaf period of ‘ZM66’ were collected. These samples were used for subsequent transcriptome sequencing and quantitative real-time PCR analysis. The remaining tea samples were dried using a freeze-dryer and then subjected to 1,2,6-TGGP content analysis. The transcriptome sequencing analysis was conducted in two biological replicates by Beijing Novogene Co., Ltd. (Beijing, China) in April 2022.

### 2.2. Measurement of 1,2,6-TGGP and Other Metabolite Contents

The 1,2,6-TGGP, caffeine, gallic acid, and components of catechins in the samples were extracted, and their contents were determined using our previously established method [13]. The dry tea sample was accurately weighed to 0.1 g and then placed in a 10 mL centrifuge tube. Then, 5 mL of a 70% methanol solution was accurately added and then extracted for 20 min in a water bath at 70 °C. After cooling to room temperature, the tea sample was transferred to a centrifuge and spun at 3500 r·min^−1^ 10 min. We aspirated 0.5 mL of the extract into a 5 mL graduated tube, added 2 mL of stabilizing solution, mixed well, and passed it through a 0.45 μm organic microporous membrane, transferred the filtrate into a sample bottle, and analyzed the content using an HPLC instrument (Waters, MA, USA). The chromatographic column selected was a Phenomenex C12, 4.6 mm × 250 mm, 5 μm (reversed-phase column), and it was operated at a temperature of 40 °C. One percent (*v*/*v*) formic acid (A) and acetonitrile (B) were used with a linear gradient (0–42 min, 4–18.7% B; 42–43 min, 18.7–4% B), and water was used as the mobile phase. These samples were injected at a flow volume of 1 mL/min and observed at 280 nm. The TGGP content was measured three times for each sample, and the standard deviation (SD) was calculated. Duncan’s multiple range test (*p* < 0.05) identified significant differences in this analysis. The standards of 1,2,6-TGGP, GA, C (catechin), EC (epicatechin), EGC (epigallocatechin), ECG (epicatechin gallate), GCG (gallocatechin gallate), GC (gallocatechin), EGCG (epigallocatechin gallate), and caffeine were purchased from Shanghai yuanye Bio-Technology Co., Ltd. (Shanghai, China) for preparing gradient concentrations to establish standard curves.

### 2.3. RNA Extraction, Library Preparation, and RNA-seq

Total RNA was extracted from one bud and one leaf of each type of tea using a Tiangen Polysaccharide Polyphenol Kit (QIAGEN, Dusseldorf, Germany). RNA quality was assessed using an Agilent 2100 Bioanalyzer (Santa Clara, CA, USA). The polyA-tailed mRNA was enriched using Oligo (dT) magnetic beads. Subsequently, the mRNA was randomly fragmented through ion interruption. The fragmented mRNA was then utilized as a template with random oligonucleotides as primers to synthesize the first and second strands of cDNA in a reverse transcriptase system. The purified double-stranded cDNA was end-repaired, A-tailed, and ligated at the sequencing junction to obtain the NEB common library. After constructing the libraries, they were initially quantified using a Qubit 2.0 Fluorometer, diluted to 1.5 ng·μL^−1^, tested for insert size using an Agilent 2100 Bioanalyzer, and then sequenced by Illumina after passing the library test. The sequencing platform used was an Illumina NovaSeq 6000 (Illumina, CA, USA). The sequencing strategy was PE150bp.

### 2.4. Data Quality Control and Mapping of Reads to the Genome

The sequenced image data were converted into raw sequence data (raw data) using CASAVA base identification and stored in the fastq format. This format contained the sequence information of the sequenced fragment and its corresponding sequencing quality information. Fastp (version 0.19.7) software was utilized for quality control to filter the sequenced sequences (reads), removing those with sequencing junctions or low-quality reads, and generating clean reads for subsequent analysis. This was done to ensure the accuracy and reliability of the data analysis. The HISAT2 (version 2.0.5) software was utilized to rapidly and accurately compare clean reads with the ‘Shuchazao’ reference genome [22] to identify the specific locations of the reads on the reference genome.

### 2.5. Identification of DEGs and Functional Enrichment Analysis

FPKM values (expected number of Fragments Per Kilobase of transcript sequence per Million base pairs sequenced) were used to represent the level of gene expression [23]. After completing the gene expression quantification, the expression data underwent statistical analysis to identify genes with significantly different expression levels in various states. Significance analysis of differential gene expression was conducted using DESeq2 (version 1.20.0) software. Differentially expressed genes between groups were identified using |log2(FoldChange)| ≥ 1.0 and padj ≤ 0.05 as the criteria. Gene Ontology (GO) functional enrichment analysis and Kyoto Encyclopedia of Genes and Genomes (KEGG) pathway enrichment analysis of the identified differentially expressed genes were conducted using the clusterProfiler (version 3.8.1) software, based on a hypergeometric distribution. Both groups used padj ≤ 0.05 as the threshold for significant enrichment.

### 2.6. Quantitative Real-Time PCR (qRT-PCR) Validation of DEGs

RT-qPCR primers were designed using Primer (version 5.0) software (Table S2) and synthesized by Zhejiang Youkang Biotechnology Co., Ltd. (Huzhou, China). The extracted RNA was reverse-transcribed to cDNA using the Tiangen FastKing cDNA First Strand Synthesis Kit and uniformly diluted to a final concentration of 50 ng·μL^−1^. GAPDH was utilized as a reference gene, and the Mona MonAmpTM ChemoHS qPCR mix reagent kit was employed for qRT-PCR analysis. The reaction system consisted of 5 μL of MonAmpTM ChemoHS qPCR mix, 0.4 μL each of forward and reverse primers, 1 μL of DNA template, and Nuclease-Free Water up to a total volume of 10 μL. The reaction procedure involved incubation at 95 °C for 10 min, followed by denaturation at 94 °C for 10 s, annealing at 58 °C for 15 s, and extension at 72 °C for 12 s, repeated for a total of 45 cycles. We performed three technical replicates and used the 2^−ΔΔCt^ method to calculate the relative expression levels of the differentially expressed genes.

## 3. Results

### 3.1. Comparison of the 1,2,6-TGGP and Related Metabolites Contents in Different Tea Samples

**Figure 1:**
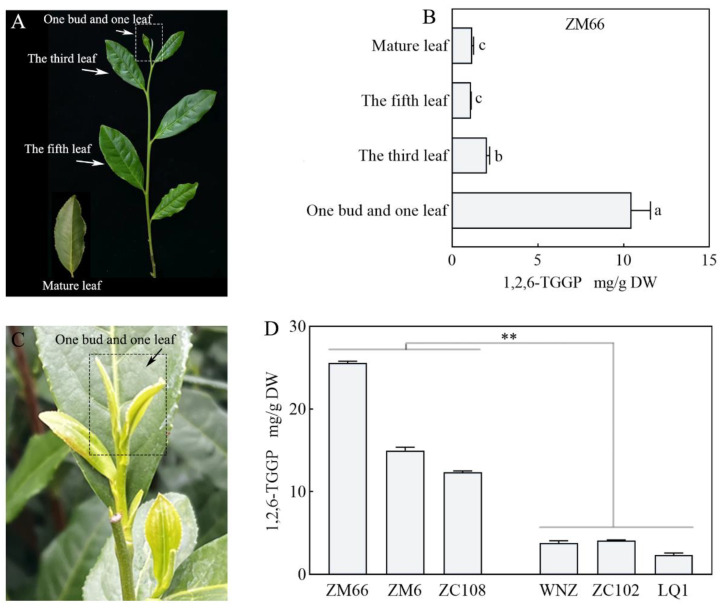
Contents of 1,2,6-TGGP in tea plants. (**A**) The sample of ‘ZM66’ at the stage of one bud and five leaves; (**B**) the contents of 1,2,6-TGGP in different leaf positions of ‘ZM66’. Different letters indicate significant differences, with *p* < 0.05. (**C**) The sample of ‘ZC108’ at the stage of one bud and one leaf; (**D**) the contents of 1,2,6-TGGP of one bud and one leaf in different tea cultivars, ** *p* < 0.01.

In 2022, to analyze the levels of 1,2,6-TGGP in various leaf positions (one bud and one leaf, the third leaf, the fifth leaf, and the mature leaf) of the tea cultivar ‘ZM66’ during the one-bud and five-leaves stage, measurements were taken (Figure 1A and Figure S1). The results showed that the 1,2,6-TGGP content in young bud leaves (one bud and one leaf) was the highest, at approximately 10.46 mg·g^−1^, and it gradually decreased with the increase in leaf positions (Figure 1B and Figure S2). Subsequently, to determine if there were variations in the 1,2,6-TGGP content in one bud and one leaf across different periods and cultivars, experimental measurements were carried out using six tea cultivars (Figure 1C,D). The 1,2,6-TGGP content of ‘ZM66’ was found to be significantly higher at the stage of one bud and one leaf compared to that of one bud and five leaves. At the same time, based on the varying levels of 1,2,6-TGGP, the six tea cultivars were categorized into two groups, with ‘ZM66’ (25.56 mg·g^−1^), ‘ZM6’ (14.98 mg·g^−1^), and ‘ZC108’ (12.37 mg·g^−1^) belonging to the high-content group, and ‘LQ1’ (2.35 mg·g^−1^), ‘WNZ’ (3.78 mg·g^−1^), and ‘ZC102’ (4.12 mg·g^−1^) belonging to the low-content group. Next, we determined the concentrations of several chemical compounds, including caffeine, gallic acid, and seven major catechins, to verify their relationships to 1,2,6-TGGP (Table 1). The findings demonstrated that these compounds in different tea cultivars exhibited large variations. However, they did not show significant differences between the high and low 1,2,6-TGGP groups, suggesting that the 1,2,6-TGGP accumulation in tea leaves is not directly affected by their biosynthesis.

### 3.2. Transcriptome Sequencing Quality Assessment and Reference Genome Alignment

As the six tea cultivars were categorized into two groups based on their 1,2,6-TGGP contents, further transcriptome analysis was performed to identify the key genes contributing to the difference. A total of twelve cDNA libraries were constructed, yielding 5.42 × 10^8^ raw reads and 81.27 GB raw bases. A total of 5.08 × 10^8^ clean reads were obtained after quality control and filtering analysis of the sequencing data. The total base number was 76.23 GB, with a GC content ranging from 43.15% to 43.98%. The Q20 value exceeded 96%, and the Q30 value exceeded 90% (Table S1). The results indicated that the sequencing quality was good.

The clean reads obtained after quality control were aligned with the reference genome of ‘Shuchazao’, and the alignment rate was higher than 83.73%. Among the reads that aligned to only position of the genome, more than 72.71% of the reads were aligned to the exon region. The results indicated that the reference genome was well annotated and suitable for use as a reference. A total of 61,546 transcripts were identified through genome alignment, and the distribution of the transcript sequence lengths was subsequently examined. Out of the total transcripts, 35,975 (58.45%) had sequence lengths that were shorter than 1200, 14,442 (23.47%) had sequence lengths ranging from 1200 to 2000, and 11,129 (18.08%) had sequence lengths that were longer than 2000. The RNA-Seq datasets have been deposited in the NCBI SRA database under accession number PRJNA935488 (https://dataview.ncbi.nlm.nih.gov/object/PRJNA935488 (accessed on 23 April 2023).

### 3.3. Screening of Differentially Expressed Genes

**Figure 2:**
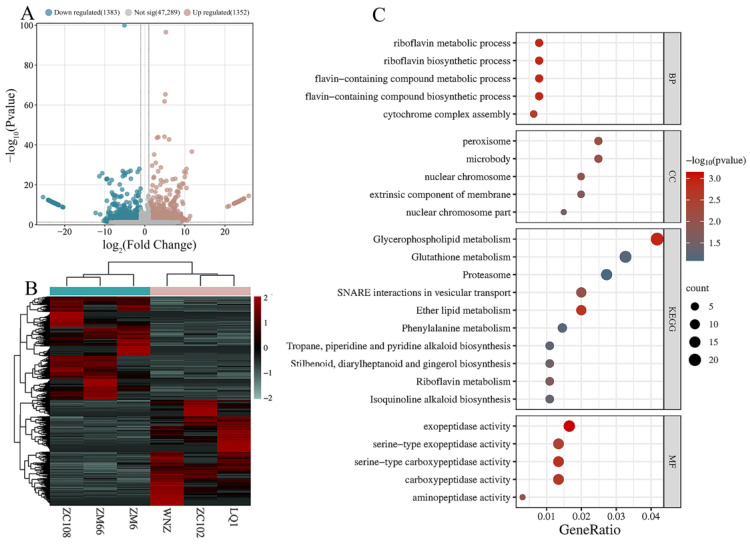
The analysis of differentially expressed genes between TGGP_H (group H) and TGGP_L (group L). (**A**) Volcano map of DEGs; (**B**) HCA of DEGs, data averaging, normalization; (**C**) the GO and KEGG enrichment pathway of DEGs.

Next, we further analyzed the gene expression differences between groups with a high and low 1,2,6-TGGP content. The original read count was used as the input file, and DESeq2 (version 1.20.0) software was employed to conduct a significant analysis of differential gene expression. The criteria |log2(FoldChange)| ≥ 1.0 and padj ≤ 0.05 were utilized to identify differentially expressed genes (DEGs). In the comparison group, a total of 2735 DEGs were identified, with 1352 genes showing significant upregulation and 1383 genes showing significant downregulation (Figure 2A). After normalizing the FPKM values of the DEGs, cluster analysis was conducted to compare the expression of the same gene in different tea samples (Figure 2B). The samples with similar expression patterns in the heat map were clustered together. Overall, there was a significant separation of differentially expressed genes between the groups, suggesting that these DEGs may be key genes influencing the 1,2,6-TGGP content.

To clarify the function of DEGs, we conducted GO and KEGG pathway functional enrichment analysis on the 2735 DEGs. Among them, 1806 DEGs were enriched in 917 GO entries, and 551 differential genes were annotated to 117 metabolic pathways. According to the *p*-value ranking, the process of riboflavin synthesis and metabolism is active and involved in various activities within the organism. Multiple genes exhibit serine carboxypeptidase activity, and several pathways related to synthesis, metabolism, and transportation are significantly enriched according to the KEGG (Figure 2C). Furthermore, it is noteworthy that the pathways that are associated with flavonoid biosynthesis ranked in the top 25% of the most significant enrichments.

### 3.4. Identification of Key Candidate Genes

**Figure 3:**
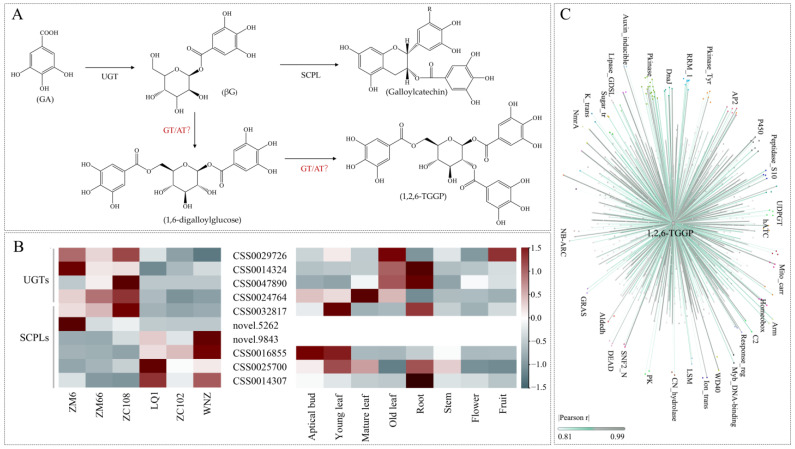
Analysis of synthesis-related genes of 1,2,6-TGGP. (**A**) Synthetic pathway of 1,2,6-TGGP. GA: Gallic acid; UGT: UDP-glucosyltransferase; βG: 1-O-galloyl-β-D-glucopyranose; SCPL: Serine carboxypeptidase-like; 1,6-digalloylglucose: 1,6-di-O-galloyl-β-D-glucopyranose; 1,2,6-TGGP: 1,2,6-tri-O-galloyl-β-D-glucopyranose; AT: acyltransferase. (**B**) HCA of genes of UGTs and SCPLs, normalization. The left figure shows the transcriptome data of this study, and the right figure shows the expression data of the ‘Shuchazao’ gene, downloaded from the internet; (**C**) correlation analysis between all DEGs and 1,2,6-TGGP, where the same color of endpoints represents the same type of genes.

The biosynthesis of 1,2,6-TGGP involves three main esterification reactions: GA → βG → 1,6-diphenylglucoside → 1,2,6-TGGP. Except for the reported involvement of UGT in the synthesis of βG, the other two steps of the reaction are still unclear (Figure 3A). According to the synthesis rules, glycosyltransferase and acyltransferase are most likely to participate in the synthesis of 1,2,6-TGGP. In the previous results (Figure 2C), the synthesis pathway of SCPL with acyltransferase characteristics has also been significantly enriched by GO. Therefore, we identified four upregulated UGTs (*CSS0029726*, *CSS0014324*, *CSS0047890*, *CSS0024764*), two upregulated SCPLs (*CSS0032817*, *novel.5262*), and four downregulated SCPLs (*CSS0016855*, *CSS0014307*, *CSS0025700*, *novel.9843*) based on their expression levels (Figure 3B).

To further identify these DEGs, we downloaded gene expression data from various tissues of the ‘Shuchazao’ genome online (Figure 3B). The previous analysis indicated that the content of 1,2,6-TGGP in one bud and one leaf of ‘ZM66’ was higher than that in mature leaves (Figure 1B). However, in ‘Shuchazao’, just four *SCPLs* were expressed at higher levels in young bud leaves than in mature leaves. Among them, only *CSS0032817* was highly expressed in cultivars with a high 1,2,6-TGGP content, while the other three genes *(CSS001685*, *CSS0014307, CSS0025700*) were all expressed at low levels. Therefore, it is proposed that *CSS0032817* has great potential for synthesizing 1,2,6-TGGP. 

Moreover, exclusively screening for anticipated genes may overlook crucial genes that have not been taken into account. To complement the above findings, we conducted a correlation analysis between 2735 DEGs and 1,2,6-TGGP. The results revealed a significant correlation between 642 genes and 1,2,6-TGGP (Figure 3C). There were 322 genes that were positively associated, including kinases that are involved in protein modification, P450 enzymes that are involved in various organic synthesis, and transcription factors such as AP2, WD40, and MYB. These findings provide the groundwork for further in-depth research.

### 3.5. Quantitative Real-Time PCR Verification of Transcriptome Data

**Figure 4:**
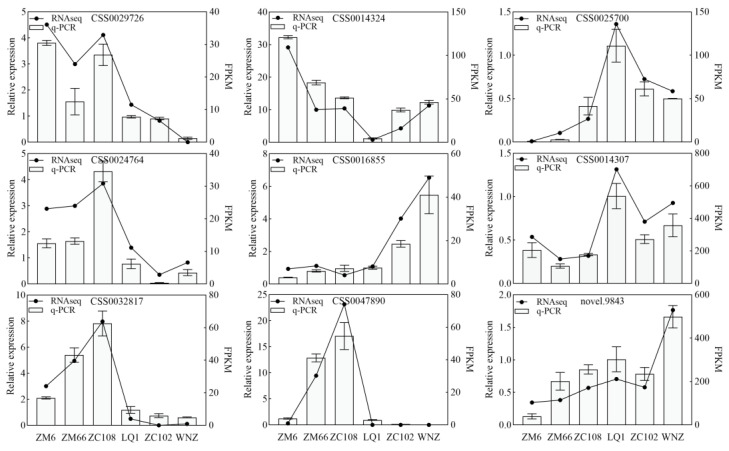
Validation of DEGs by qRT-PCR.

According to the biosynthetic pathway of 1,2,6-TGGP, *UGTs* and *SCPLs* play important roles, and 10 related genes were identified in the DEGs (Figure 3). To further validate the reliability of the transcriptome sequencing results, we selected the 10 DEGs for qRT-PCR. Except for the low expression level of *novel.5262* that was not accurately detected, the expression of the other nine genes was consistent with the transcriptome sequencing results (Figure 4). This indicates that the results of this transcriptome analysis are reliable.

## 4. Discussion

Tea is a popular beverage worldwide, thanks to its high levels of secondary metabolic components. Among these metabolites, 1,2,6-TGGP is a newly discovered compound in tea plants, and the genetic mechanism associated with its accumulation is not yet clear. Zhang et al. (2013) analyzed 16 tea samples and discovered that the 1,2,6-TGGP content in the samples was low, ranging from 0 to 6.6 mg·g^−1^ [24]. In our previous study, we measured the 1,2,6-TGGP content in 17 tea cultivars, which exhibited a wide variation, ranging from 1.96 to 43.20 mg·g^−1^ [13]. Subsequently, we identified additional tea cultivars over several consecutive years and discovered that the relative content of 1,2,6-TGGP remained consistent across different cultivars and years. Previous studies have indicated that the concentration of 1,2,6-TGGP decreases over time with harvesting [13]. This experiment once again confirms the previous result, and the peak content time was found to occur at the stage of one bud and one leaf in spring (Figure 1). To identify the key candidate genes affecting the accumulation of 1,2,6-TGGP in *Camellia sinensis*, we selected one bud and one leaf from six tea cultivars with stable and significant differences in 1,2,6-TGGP content for transcriptome sequencing analysis. A total of 2735 DEGs were identified (Figure 2). GO and KEGG analyses, combined with the synthetic pathway for 1,2,6-TGGP (Figure 3A), revealed the complexity of the 1,2,6-TGGP synthetic pathway, suggesting the potential involvement of both UGTs and SCPLs. 

UDP Glycosyltransferases (UGTs) are enzymes that facilitate the transfer of glycosyl groups from an activated donor molecule to a specific acceptor molecule, leading to the formation of a wide variety of glycosidic compounds [25]. UGT primarily utilizes small-molecule compounds, such as flavonoids and phenolic acids, as glycosyl acceptors, and UDP-glucose and UDP-galactose as glycosyl donors [26]. Glycosylation alters the hydrophilicity of the receptor molecule, enhancing its solubility, stability, metastability, and diversity. In addition, glycosylation contributes to the storage and transport of acceptors within cells and organisms, ultimately affecting their ability to perform biological functions [27,28]. Their primary function is to regulate the synthesis of secondary metabolites in plant secondary metabolism [28]. In this study, a total of four upregulated differentially expressed UGTs were identified, including 7-deoxyloganetin glucosyltransferase-like (*CSS0047890*), hydroquinone glucosyltransferase-like (*CSS0024764*), anthocyanidin 3-O-glucosyltransferase 2-like (*CSS0014324*), and beta-D-glucosyl crocetin beta-1,6-glucosyltransferase-like (*CSS0029726*). A further analysis revealed that they were not correlated with the 1,2,6-TGGP content in different tea tissues (Figure 3B,C). There have been no reports indicating the potential catalytic activity of gallic acid. In addition, previous studies have shown that *CsUGT84A22* (*CSS00043088*) is a gene that regulates the key enzyme that is involved in βG synthesis. The gene’s expression level is closely related to the 1,2,6-TGGP content, but not to the catechin content [13,18,29]. During this experiment, as the sample size increased, *CsUGT84A22* did not exhibit any intergroup differences. This indicates that as a common precursor of gallocatechins and gallotannins, βG, is not only highly expressed in varieties with high levels of 1,2,6-TGGP, which may be influenced by multiple factors. In conclusion, further investigation is needed to understand the accumulation mechanism of UGTs in 1,2,6-TGGP.

SCPLs are proteins with high structural and functional similarity to serine carboxypeptidases (SCP), and both are members of the peptidase_S10 superfamily with a conserved pfam00450 domain [30,31]. SCPLs are involved in a variety of biological processes, including plant growth and development, brassinosteroid signaling transduction, the abiotic stress response, and secondary metabolism [32]. Some SCPLs have also evolved to function as acyltransferases. Two major families of acyltransferases use phenolic compounds as acceptor or donor molecules, each using a distinct type of acyl donor. The majority of the identified acyltransferases belong to the *BAHD* acyltransferase family, which is characterized by using acyl-CoA thioesters as donor molecules. SCPL uses βG as an acyl donor to participate in the secondary metabolism of plants [20]. In tea plants, the enzyme *ECGT*, which catalyzes the acylation of βG to produce ester-type catechins, belongs to the *SCPL* family [33]. Recent research has demonstrated that SCPL can catalyze the formation of the corresponding ester catechins of EGCG andECG from EGC and EC using 1,2,3,4,6-penta-O-galloyl-D-glucose (PGG) or βG as an acyl donor [20]. 1,2,6-TGGP is structurally similar to βG and PGG, which belong to hydrolyzable tannins. Therefore, SCPLs are also possibly invotlved in the conversion of 1,2,6-TGGP to EGCG or ECG. 

Based on this, we screened two upregulated *SCPLs* that may be involved in synthesis and four downregulated *SCPLs* that may be involved in metabolism (Figure 3B). The functions of the collateral homologs of SCPL-AT vary. Ahmad et al. (2020) conducted a genome-wide analysis of the serine carboxypeptidase-like acyltransferase gene family in tea plants and identified SCPL-IA as having potential acylation functions [20,29]. Additionally, the differentially expressed gene *CSS0016855* was classified as SCPL-II. *CSS0032817*, a member of SCPL-IA, is highly accumulated in tea plants with low levels of ECG and EGCG and is believed to be unrelated to the galloylation modification of catechins [34]. In this experiment, after comparing the gene expression levels between different varieties and tissues, we found that only *CSS0032817* exhibited the trend of changes in 1,2,6-TGGP. Therefore, we speculate that *CSS0032817* may be involved in regulating the final two steps of the 1,2,6-TGGP synthesis pathway. 

So far, all the analyses in this paper have focused on the enzyme genes UGT and SCPL, and the anabolic metabolism of one metabolite requires multi-party cooperation. The high accumulation of 1,2,6-TGGP enhances the activity of certain secondary metabolic pathways. To explore the potential of other genes, we conducted a correlation analysis of 2735 differentially expressed genes. Several genes were found to be significantly associated with 1,2,6-TGGP, including cytochrome P450 and various transcription factors (Figure 3C). Studies have shown that P450 is involved in the synthesis of oat acylation molecules and is closely related to the position of the *SCPL17*, which directly mediates the acyltransferase reaction [35,36]. In *Camellia*, P450 is significantly positively correlated with galloylated catechins [37]. Transcription factor genes, such as MYB and WD40, are also involved in the catechin synthesis pathway. Among them, *PgMyb308-like* gene can inhibit the synthesis of hydrolyzable tannins in *Punica granatum* L. [38]. It is evident that the anabolic pathway of substances is complex, and further investigation is needed to understand the specific accumulation mechanism of 1,2,6-TGGP in the future. 

In conclusion, this study has preliminarily identified the gene *CSS0032817* of serine carboxypeptidase-like acyltransferase to be associated with 1,2,6-TGGP accumulation based on transcriptome sequencing. This represents the first attempt to molecularly elucidate the mechanisms affecting 1,2,6-TGGP accumulation, and further research is needed to study the function of the gene. Taken together, the results provide insight into the molecular mechanisms of 1,2,6-TGGP accumulation.

## Figures and Tables

**Table 1:** Contents of caffeine, gallic acid, and catechins in 6 tea cultivars.

	ZM6	ZM66	ZC108	ZC102	WNZ	LQ1
GA	7.54 ± 0.09 e	8.42 ± 0.10 c	9.40 ± 0.10 a	9.07 ± 0.04 b	7.90 ± 0.12 d	5.66 ± 0.07 f
GC	3.95 ± 0.15 a	0.96 ± 0.29 bc	1.02 ± 0.06 bc	0.83 ± 0.11 c	0.45 ± 0.26 c	1.34 ± 0.23 b
EGC	1.11 ± 0.05 a	0.87 ± 0.02 b	0.99 ± 0.02 a	0.59 ± 0.09 cd	0.42 ± 0.06 d	0.61 ± 0.02 c
C	1.60 ± 0.28 e	5.61 ± 0.10 b	9.17 ± 0.09 a	2.41 ± 0.03 c	2.11 ± 0.08 d	1.47 ± 0.00 e
EC	6.39 ± 0.42 a	5.30 ± 0.31 b	4.68 ± 0.03 c	4.35 ± 0.05 d	5.37 ± 0.22 b	6.92 ± 0.14 a
GCG	0.74 ± 0.03 a	0.32 ± 0.16 c	0.65 ± 0.01 b	0.39 ± 0.02 c	0.36 ± 0.18 c	0.72 ± 0.03 ab
ECG	0.58 ± 0.07 b	0.20 ± 0.03 c	1.53 ± 0.00 a	1.54 ± 0.05 a	1.62 ± 0.28 a	0.20 ± 0.15 c
EGCG	68.87 ± 0.1.86 a	60.67 ± 0.52 b	69.15 ± 0.87 a	66.19 ± 1.54 a	55.74 ± 2.19 c	58.83 ± 1.36 b
Caffeine	25.44 ± 0.40 b	31.79 ± 0.40 a	31.87 ± 0.36 a	31.29 ± 0.16 a	30.92 ± 0.41 a	27.62 ± 0.34 b

The data are the average ± standard deviation, and the content unit is mg/g. Different lowercase letters indicate significant differences.

## Data Availability

Data is contained within the article or Supplementary Material.

## Supplementary Materials

The following supporting information can be downloaded at https://www.mdpi.com/article/10.3390/foods13030495/s1: Figure S1: Chromatogram in different leaf positions; Figure S2: Chromatogram in different tea cultivars; Table S1: Sample sequencing data, evaluation statistics, and genome alignment; Table S2: Genes and primers for qPCR.
