# Supplementary material for: Identification of Key Genes Associated with 1,2,6-Tri-O-galloyl-β-D-glucopyranose Accumulation in Camellia sinensis Based on Transcriptome Sequencing
Source: Foods. 2024 Feb 4;13(3):495. doi: 10.3390/foods13030495 (PMC10855904; doi:10.3390/foods13030495)
Supplement: Supplementary file 1 [file foods-13-00495-s001.zip › foods-2840306-supplementary.pdf]

Supplementary Figure S1. Chromatogram in different leaf positions

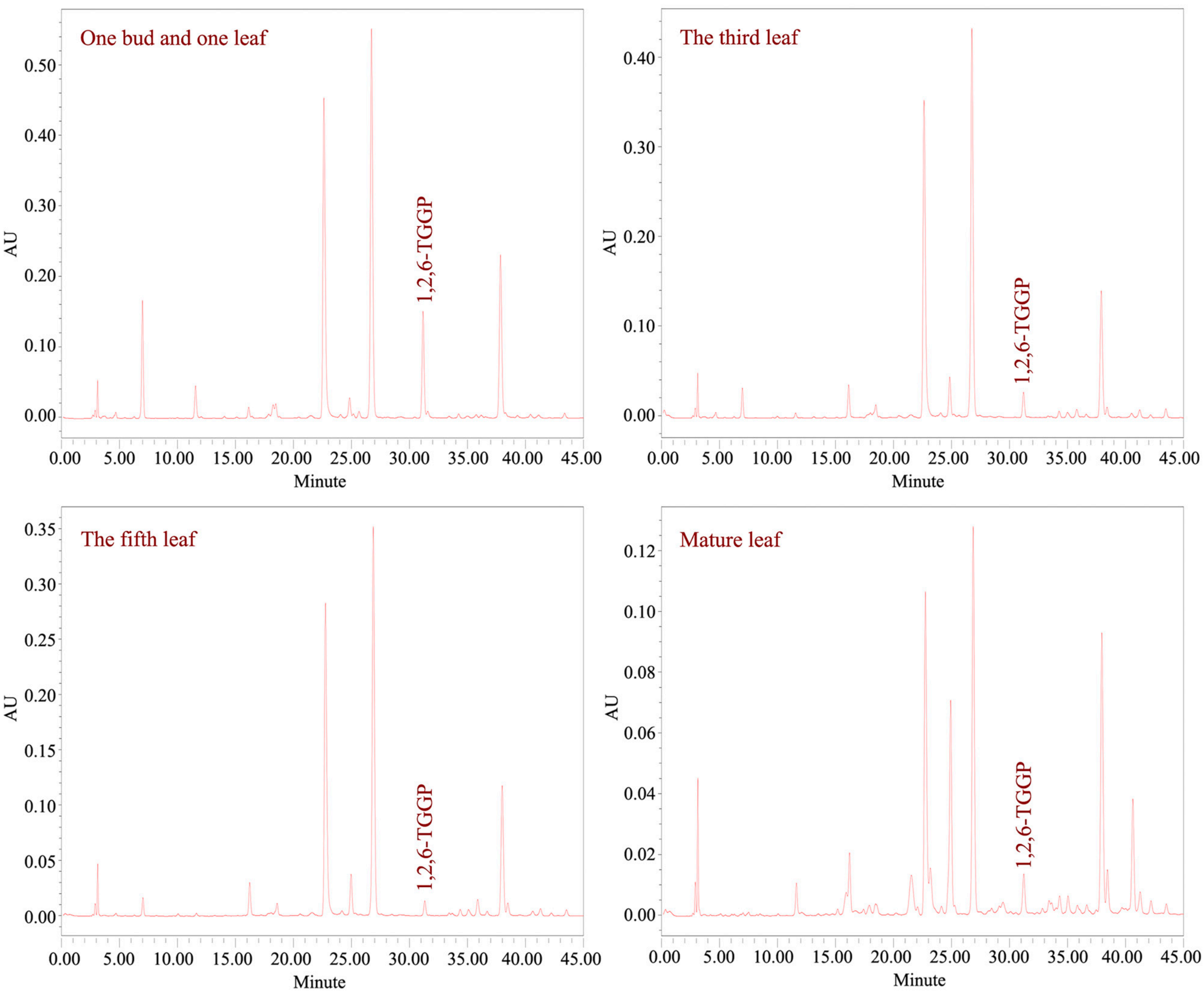

Supplementary Figure S2. Chromatogram in different tea cultivars

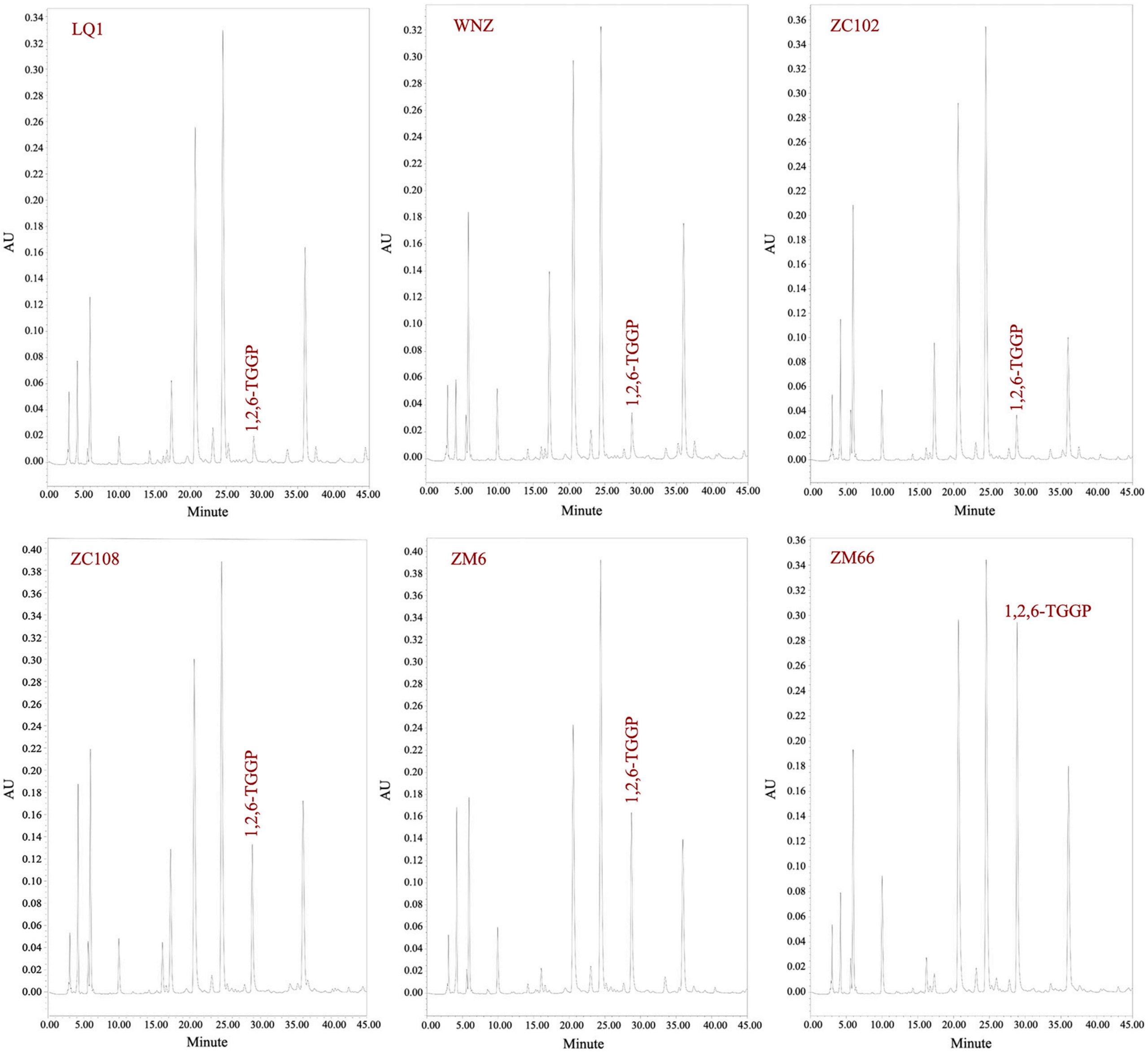

Supplementary Table S1. Sample sequencing data evaluation statistics and genome alignment

Sample	Raw reads	Clean bases(G)	Error rate (%)	Q20(%)	Q30(%)	GC content (%)	Total mapping (%)	Mapped to exon (%)
ZM66_1	44289986	6.02	0.03	96.56	90.81	43.15	84.94	73.32
ZM66_2	45208290	6.19	0.03	96.83	91.35	43.55	85.95	76.12
ZM6_1	41461790	6.21	0.03	96.81	91.38	43.92	84.72	77.13
ZM6_2	45354704	6.19	0.03	96.76	91.27	43.51	84.60	75.64
ZC108_1	47952532	7.19	0.03	96.76	91.20	43.77	85.53	77.08
ZC108_2	44362804	6.06	0.03	96.71	91.07	43.46	85.63	73.86
ZC102_1	44970534	6.74	0.03	96.52	90.67	43.47	83.73	73.74
ZC102_2	48219098	6.69	0.03	97.02	91.80	43.8	84.93	75.86
WNZ_1	47292262	6.59	0.03	97.18	92.1	43.67	85.70	76.24
WNZ_2	44577326	6.06	0.03	96.7	91.1	43.98	85.22	77.92
LQ1_1	42907800	6.04	0.03	96.81	91.29	43.96	85.40	76.68
LQ1_2	45254570	6.25	0.03	96.81	91.31	43.3	84.85	72.71

Q20: The percentage of bases with Phred values greater than 20 in total bases; Q30: The percentage of bases with Phred values greater than 30 in total bases

Supplementary Table S2. Genes and primers for qPCR

Gene ID	Forward primer (5′-3′)	Reverse primer (5′-3′)
CSS0024764	GTCCACGGTGTGCCTCTAAT	CCTTCCCATCTTCTCCTTCC
CSS0047890	CACGACGTAGCTGCTCTCTG	AAAACCCATGAAGCCACAAG
CSS0014324	TCGATAACAAGGGGACAAGC	AATCACAAACCCAGCGAGTC
CSS0029726	CGGTGGTTAGTTTTGGCAGT	GTGATGTTGCAAGGTGGTTG
CSS0032817	CACAAGGGAGACAAAATGAAG	ATCCGATGGAGGAGGAGAAAGA
CSS0025700	GGCGAGCATACCCTTTGTAG	GGTTCGATGCCAACTTCATT
CSS0014307	TCAGCGCCAAAGGTTATAGG	CCTGCTCCCTTGACAGTAGC
CSS0016855	CCAAAAGGCATCGAATGAGT	TCTGCACATCTGGCCTATTG
novel.5262	CCGCATCCATCGACTTTTAT	AGCCCCTCTGGAGAGAAGAC
novel.9843	CCATGTTCCGGTGTAGGTCT	TCAACGGTCACATACCCAGA
GAPDH	TTGGCATCGTTGAGGGTCT	CAGTGGGAACACGGAAAGC
